# Nutritional counseling in childhood and adolescence: a systematic review

**DOI:** 10.3389/fnut.2024.1270048

**Published:** 2024-02-01

**Authors:** Lenycia de Cassya Lopes Neri, Monica Guglielmetti, Simona Fiorini, Federica Quintiero, Anna Tagliabue, Cinzia Ferraris

**Affiliations:** ^1^Laboratory of Food Education and Sport Nutrition, Department of Public Health, Experimental and Forensic Medicine, University of Pavia, Pavia, Italy; ^2^Human Nutrition and Eating Disorder Research Center, Department of Public Health, Experimental and Forensic Medicine, University of Pavia, Pavia, Italy

**Keywords:** nutritional counseling, children, adolescents, nutritional strategies, systematic review

## Abstract

Healthy eating habits are the basis for good health status, especially for children and adolescents, when growth and development are still ongoing. Nutrition educational programs are essential to prevent and treat chronic diseases. Nutritional counseling (NC), as a collaborative process between the counselor and the client process, could help to achieve better outcomes. This review aims to collect information about the utilization of NC during childhood and adolescence and to highlight its possible impact on adherence/compliance rates, nutrition knowledge, status and dietary intake. The methods applied in this systematic review followed the instruction of the Preferred Reporting Items for Systematic Reviews and Meta-Analyses (PRISMA). The search in PubMed/Medline, Scopus, Web of Science, LILACS, and Science Direct included observational or randomized studies. RoB 2.0 and Robins-I tools was used for the risk of bias assessment in randomized and non-randomized studies, respectively. The quality of evidence was checked by the Mixed Methods Appraisal Tool system. A total of 21 articles were selected, computing 4,345 individuals. 11 achieved at least 4 stars quality level. The highest risk of bias for randomized studies was related to the randomization process. 42.9% of non-randomized studies had some concerns of bias, mainly because of a lack of control of all confounding factors. Different strategies of NC were used in children and adolescents with positive results for health or diseases. NC strategies can be effectively used in children and adolescents. In general, NC showed benefits in pediatrics age for anthropometric or body composition parameters, dietary intake, nutrition knowledge and physical activity improvement. Performing NC in pediatrics is challenging due to the counseling strategies that must be adapted in their contents to the cognitive ability of each age. More structured research must be done focused on this population. Investments in healthy eating behaviors in pediatrics can lead to better health outcomes in the future population with substantial benefits to society.

**Systematic review registration:**

[https://www.crd.york.ac.uk/prospero/#recordDetails], identifier [CRD42022374177].

## 1 Introduction

According to a recent publication from United Nations International Children’s Emergency Fund (UNICEF) and the World Health Organization (WHO) and the World Bank Group malnutrition is spread among children worldwide. In particular, 144 million children under 5 years-old were classified as stunting, 47 million as wasted and 38.3 million were overweight (1). In 2017, the Joint Food and Agriculture Organization (FAO) and WHO expert committee on food additives reported that less than half of pediatric rural communities worldwide meet the dietary requirements for any food group (2). Thus, globally, children are not achieving the dietary recommendations.

The dietary patterns and eating behavior have important implications, especially for preventing chronic diseases or syndromes, such as metabolic syndrome, cardiovascular diseases, diabetes, cancers and chronic respiratory diseases (3).

Eating habits are considered modifiable behavioral risk factors for chronic diseases. A good eating habit, as part of a lifestyle, is among the multiple interacting factors of an overall good health status. The two main contributing factors are (i) biological (internal influence, for example, genetic predisposition) and (ii) psychosocial aspects (external or environmental influence) (2). These factors must be considered when choosing an intervention for delivering nutritional care (4). Special attention must be given during childhood and adolescence, when nutritional intervention could favor a voluntary adoption of healthier eating habits and behaviors, leading to good health and quality of life (5). In these phases, the importance of studying good instruments of intervention increases, in order to face the challenges experienced during childhood (parental habits, family adherence) and adolescence (emotional and psychological changes during this phase).

Appropriate care and optimal feeding practices during the pregnancy and first 2 years of life, considered the first 1,000 days, can also prevent undernutrition and reduce morbidity and mortality during childhood worldwide. Nutrition interventions in this phase could be considered a great opportunity to construct health benefits even during adulthood (6). Adolescence is also a particular life phase marked by physical and social changes and the development of individuality and identity (7). There are some nutritional difficulties such as delivering a healthy diet and the eminence of the burden of obesity or eating disorders. Optimal nutrition is also essential to improve outcomes in children with diseases, in order to establish and maintain good health. It is essential to deliver nutritional information in a way to change lifestyles and produce health outcomes in the long term (8).

Nutritional counseling (NC) is a process of collaboration between the counselor (i.e., the healthcare professional) and the client (i.e., the patient) to establish priorities, goals, and action plans in nutrition and physical activity (4). Only a few articles included a whole perspective of applying the theory or model of NC (such as Cognitive Behavioral Theory, Transtheoretical Model, Social Cognitive Theory) directly for the pediatric population. These theories support several strategies, for example, self-monitoring, problem solving, motivational interviewing, goal setting and others (4).

Recent reviews supported the use of NC (9) in healthy athletes (10) or in cases of diseases, such as cancer (11) or other chronic conditions (4). To the best of our knowledge, no publications reviewed the use of NC in the pediatric age group.

This review aims to collect information about the utilization of NC during childhood and adolescence and to highlight its possible impact on adherence/compliance rates, nutrition knowledge, status and dietary intake.

## 2 Materials and methods

The methods applied in this systematic review followed the instruction of the Preferred Reporting Items for Systematic Reviews and Meta-Analyses (PRISMA). PubMed/Medline, Scopus, Web of Science, LILACS, and Science Direct were used to carry out the articles search. The languages allowed were English, Italian, Portuguese, and Spanish, according to the capability of comprehension of the authors. No time limit was considered since no reviews had been made with this approach before.

Interventional trials, observational studies, case reports, and case series were included, independently of whether they were controlled, randomized or not. Exclusion criteria were: full text unavailable, manuscripts without the outcomes of interest; reviews, opinion articles, guidelines, letters, editorials, comments, news, conference abstracts, theses, dissertations, and *in vitro* or animal studies. Articles regarding the children’s health in which the addressees of the NC were adults or elderly were also excluded.

### 2.1 Literature search

The terms used for the electronic search were “Nutritional counseling” OR “Nutritional counseling” OR “Nutritional and eating education” OR “Nutritional program,” combined with the terms “Compliance,” “Guideline Adherence,” “Patient Compliance,” OR “Treatment Adherence and Compliance.” The final search was built as shown in Table 1. In case of recommendation from experts, gray literature was searched using Google Scholar and the articles were manually included. The population involved in this review was restricted to pediatric age (0–18 years old) and the comparison was any traditional dietary advice strategy. Detailed criteria for inclusion and exclusion are described in Table 2.

**TABLE 1:** Search strategy according to the database and numeric initial results in number of articles.

Data base	Search strategy	Number of articles
PubMed	{(“Nutritional counseling”[All Fields] OR “Nutritional counseling”[All Fields] OR “Nutrition Program”[All Fields] OR “Nutritional education”[All Fields] OR “EATING education”[All Fields]) AND (“Compliance”[MeSH Terms] OR “Guideline Adherence”[MeSH Terms] OR “Patient Compliance”[MeSH Terms] OR “Treatment Adherence and Compliance”[MeSH Terms])} AND (allchild[Filter])	77
Scopus	(“Nutritional counseling” OR “Nutritional counseling” OR “Nutrition Program” OR “Nutritional education” OR “EATING education”) AND (“Compliance” OR “Guideline Adherence” OR “Patient Compliance” OR “Treatment Adherence and Compliance”) AND (“CHILD”)	135
Web of Science	(“Nutritional counseling” OR “Nutritional counseling” OR “Nutrition Program” OR “Nutritional education” OR “EATING education”) AND (“Compliance” OR “Guideline Adherence” OR “Patient Compliance” OR “Treatment Adherence and Compliance”) AND (“CHILD”)	16
Lilacs	(“Nutritional counseling” OR “Nutritional counseling” OR “Nutrition Program” OR “Nutritional education” OR “EATING education”) AND (“Compliance” OR “Guideline Adherence” OR “Patient Compliance” OR “Treatment Adherence and Compliance”) AND (“CHILD”)	2
Science Direct	(“Nutritional counseling” OR “Nutritional counseling” OR “Nutrition Program” OR “Nutritional education” OR “EATING education”) AND (“Compliance” OR “Treatment Adherence and Compliance”) AND (“CHILD”)	1,524

**TABLE 2:** PICOS criteria of inclusion and exclusion.

PICOS criteria	Inclusion criteria	Exclusion criteria
Population	Children and adolescents	Adults, elderly
Intervention	Nutritional counseling strategies	No nutritional intervention
Comparison	Traditional dietary advice	Not applicable
Outcomes	Adherence/compliance rates/nutrition knowledge/dietary intake/nutritional status	Not applicable
Types of studies included	Randomized controlled trials; uncontrolled observational studies; case reports and case series. In case of recommendation from experts, articles were manually included.	Full text not available; opinion articles, conference abstracts, theses, and dissertations; without the outcomes of interest; reviews, guidelines, letters, editorials, comments, news, and *in vitro* or animal studies
Research question	What information is available in literature about the utilization of nutritional counseling during childhood and adolescence?	

### 2.2 Study selection

The discrimination of the included or excluded articles was independently carried out by two authors (LN and SF). The process of study selection was carried out using the Rayyan software (12), following three steps: 1–reading the titles and abstracts; 2–evaluation of the full text articles selected and 3–if relevant, inclusion of other studies present in the references of the selected articles. The study selection process was based on the PICOS strategy [Population (P): children and adolescents, Intervention (I): nutritional counseling strategies, Control (C): standard dietary treatment, Outcome (O): adherence/compliance rates/nutrition knowledge, Study type (S): randomized controlled trials; uncontrolled observational studies; case reports and case series]. These were considered the inclusion criteria to select titles and abstracts. All the potentially relevant abstracts were obtained in full-text version in order to verify the inclusion. In case of disagreement between the two authors during the blind process of selection, a third author analyzed the full-text articles for the final decision (CF). Subsequently, some articles were added manually, as they were relevant to the search. The selected studies were included in the qualitative analysis.

The risk of bias of each selected article was evaluated by two authors independently (LN and FQ) using the RoB 2.0 Cochrane tool for randomized studies (13). Five domains were analyzed: (1) randomization process, (2) deviations from intended interventions, (3) missing outcome data, (4) measurement of the outcome, and (5) selection of the reported result(s). The Robins-I tool (14) was used for non-randomized studies, checking 7 domains at pre-intervention (bias due to confounding, or in the selection of participants into the study), intervention (bias in classification of interventions) and post-intervention (biases due to: deviations from intended interventions, missing data, measurement of outcomes, and selection of the reported result). Funding was analyzed in order to detect possible bias in publication (Supplementary Material 1).

Beyond the risk of bias evaluation, the quality of evidence was also checked by two authors independently (LN and FQ) using the Mixed Methods Appraisal Tool (MMTA) system (version 2018) (15). The decision of discrepancies was made by a third author (SF). Data about studies’ samples characteristics, design, intervention, results, and quality were extracted and presented in tables.

## 3 Results

**FIGURE 1:**
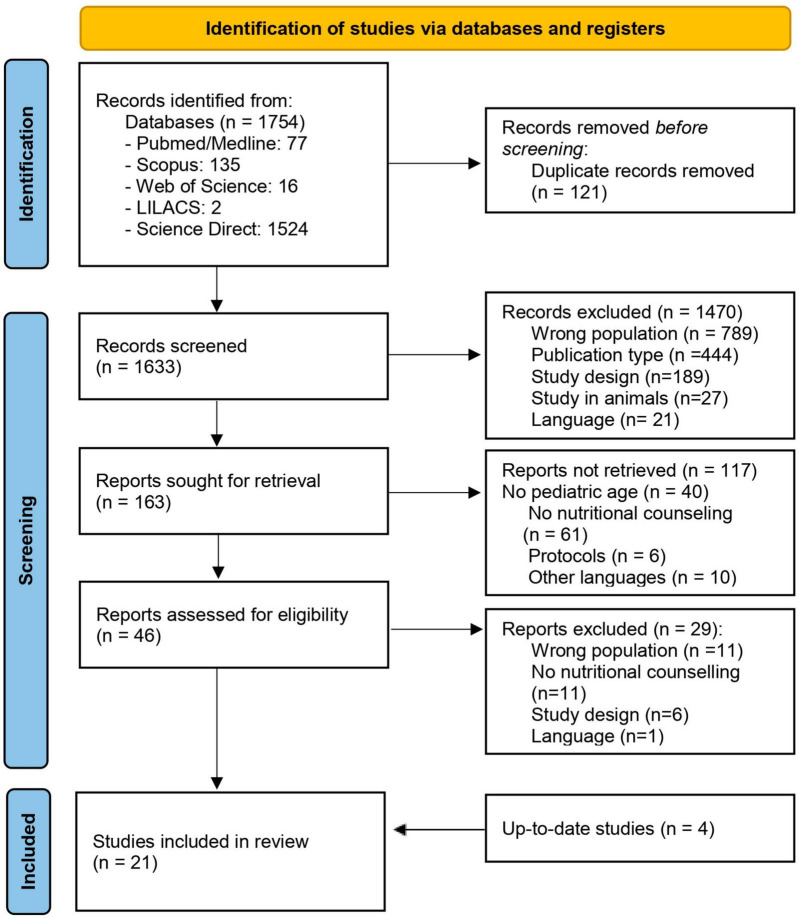
Flowchart of the selection process for identified records from databases. From: Page et al. (59).

The search strings were applied to various databases, obtaining 1,754 results. The selection process and the number of articles retained at each stage are described in the PRISMA flowchart (Figure 1). Following this process, 21 articles were selected, including a total of 4,345 pediatric patients (sample size ranged from 7 to 1,159). Studies in which NC was not directed to children or adolescents (16–20) or the intervention was not truly NC (21–26) were excluded. Further details for each of the chosen articles are outlined in Table 3, in which the studies are grouped according to the absence (27–33) or presence of randomization (27–46). Two articles (32, 46) were classified as randomized trials because there is randomization mentioned in methods, despite these being classified as non-randomized trials in the registration into the clinical trials platform.

**TABLE 3:** Characteristics of selected articles.

References, country	Type of study	Sample	Intervention	Results	Risk of bias (Rob2/Robins)	Quality (MMAT)
**Randomized studies**						
(34), USA	Randomized controlled trial	*n* = 8 children with cystic fibrosis Age, y (mean ± SD): 7 ± 1.7 y (intervention) 6 ± 2.7 y (control) Weight for age, percentile (mean ± SD): 18th ± 9.7 (intervention) 9th ± 4.8 (control)	6 weeks behavioral intervention (*n* = 5) Control: waiting list (*n* = 3) Follow-up: n.d. Professionals: clinical and pediatric psychologist, dietitian	Behavioral group: ↑ caloric intake (*p* = 0.03) ↑ weight (*p* = 0.03) No changes on pulmonary function and resting energy expenditure or activity level Adherence/compliance rates: n.d.	High	***
(47), USA	Randomized controlled trial	*n* = 7 children with cystic fibrosis Age, y (mean): 10 y Weight for age, percentile (mean): 12th	Five 90-min treatment sessions on a weekly basis; 2 groups: Behavioral intervention: behavior-management strategies Nutrition education: no behavioral strategies Follow-up: 2 years Professionals: pediatric psychology, pediatric dietitian	Behavioral intervention vs. nutrition education: ↑ daily caloric intake ↑ weight Improved caloric intake was maintained 2 years following treatment Adherence/compliance rates: no changes on rating for the adherence or behavioral compliance scale.	High	****
(35), USA	Randomized controlled trial	*n* = 57 adolescents with prehypertension or hypertension Age, y (mean ± SD): 14.3 ± 2.1 (DASH); 14.4 ± 2.1 (RC) Weight for age, percentile (mean): n.d.	60-min face-to-face counseling for 3-month: behavioral nutrition intervention DASH diet vs. routine care (RC) (control): Follow-up: 6 months Professionals: dietitian	DASH compared to RC had: ↓ SBP z scores from baseline to post-treatment (*p* < 0.01) ↓ SBP z scores from baseline through follow-up (*p* < 0.1). ↑ intake of fruits (*p* < 0.001), potassium and magnesium (*p* < 0.01) ↓ total fat (*p* < 0.05) from baseline to post-treatment ↑ low fat dairy (*p* < 0.001) from baseline through follow-up Adherence/compliance rates: n.d.	Low	****
(36), USA	Randomized controlled trial	*n* = 22 overweight female students Age, y (mean ± SD): 10.5 ± 0.8 (soccer); 10.3 ± 1.0 (control) BMI for age, percentile (mean) > 97th	Social Cognitive Theory 2 months of recreational soccer (*n* = 14) or Control: waiting list control (*n* = 8) Follow-up: 5 months Professionals: chiropractic physician	Both group: ↑ nutrition knowledge (*P* < 0.002) No association between nutrition knowledge and follow-up BMI (*r* = -0.185; *p* = 0.462) Adherence/compliance rates: n.d.	High	***
(37), Netherlands	Randomized controlled trial	*n* = 122 obese adolescents Age, y (mean ± SD): 14.5 ± 1.7 (intervention); 14.4 ± 1.8 (control) BMI, kg/m^2^ (mean ± SD): 33.3 ± 4.6 (intervention); 33.6 ± 5.1 (control) BMIsds: 2.93 ± 0.41 (intervention); 2.93 ± 0.51 (control)	Intervention: Cognitive Behavioral Therapy; 7 sessions (90 min) with an interval of 2–3 weeks Control: standard treatment Follow-up: 18 months Professionals: dietitian, pediatricians/endocrinologist and psychologist	Intervention group: ↓ BMI at 18 months (*p* < 0.05) Adherence/compliance rates (> 5 sessions): 59.2%	Some concerns	****
(38), Brazil	Paired cluster randomized school-based trial	*n* = 559 students (intervention: *n* = 277, control: *n* = 282) Age, y (mean ± SD): 11.2 ± 1.3 BMI, kg/m2 (mean ± SD): 17.4 ± 3.0 (intervention); 18.6 ± 3.7 (control)	9 months intervention: monthly 1-h classroom sessions included playing games, staging of theater sketches, watching movies and puppet shows, and writing and drawing contests. Follow-up: n.d. Professionals: trained nutritionists	Intervention group: ↓ sugar-sweetened beverages and cookies ↑ fruits NS: changes in BMI between the 2 groups (*p* = 0.003; *p* = 0.75). Adherence/compliance rates: measured as frequency of food consumption *p* < 0.01	Some concerns	****
(39), USA	Randomized controlled trial	*n* = 40 overweight/obese adolescents (intervention) *n* = 21 historical patients (control) Age, y (mean ± SD): 15.4 ± 1.8 (intervention); 15.7 ± 1.5 (control)	Transtheoretical Model 7-min DVD + 20–30 min of standardized verbal and written nutrition education Follow-up: 4–6 weeks Professionals: registered dietitian nutritionist and physician or nurse practitioner	Intervention group: ↑ parents’ knowledge of obesity-related comorbidities. NS: weight-related outcomes in this adolescent clinic. Adherence/compliance rates: n.d.	Low	**** *
(40), Italy	Cluster randomized controlled trial	*n* = 389 children Age, y (mean ± SD): 3.4 ± 0.1 BMI, kg/m^2^ (mean ± SD): 16.2 ± 0.07 BMI z-score, kg/m^2^ (mean ± SD): 0.28 ± 0.03	Intervention: 6-month-long Two face-to-face motivational interviews with parents; learning experiences with children about healthy behaviors Control: usual care Follow-up: 2 years Professionals: nurses, primary care pediatricians, teachers	Intervention group compared to control group: 48.4% of children showed a low-risk of combined health behavior score 4 energy-related behaviors in the medium and long term successfully changed: FV intake, physical activity, TV-watching time and SSB intake Beneficial changes in target behaviors and CHBS in intervention children whose mothers had a medium/high level of education NS: BMI outcomes between groups. Adherence/compliance rates: n.d.	Low	****
(41), USA and Canada	Randomized clinical, parallel-group study	*n* = 38 overweight/obese adolescents Age, y (mean ± SD): 14.1 ± 1.7 (intervention); 15.7 ± 1.4 (control) BMI, kg/m^2^ (mean): 32.0 (intervention); 31.9 (control)	6-month of weight-reducing diet: individual nutrition education and behavioral counseling (in-person sessions and telephone counseling calls; 6 total contacts) + daily text messages Intervention group: standardized weight-reducing diet + water advice Control group: standardized weight- reducing diet Follow-up: n.d. Professionals: registered dietitian	In intervention group: ↑ self-reported water intake at 6 months (*p* < 0.001) Adherence/compliance rates: lack of adherence.	Low	**** *
(42), USA	Cluster-randomized controlled	*n* = 1,159 students Age, y (mean ± SD): 10.6 ± 0.6 BMI, kg/m^2^ (mean ± SD): n.d. BMI z-score (mean ± SD): 0.7 ± 1.2	4 groups: – Curriculum: 23 science lessons based on social cognitive and self-determination theories – Wellness: food policy and physical activity bouts of Dance Breaks – Curriculum + wellness – Control Follow-up: n.d. Professionals: teachers	Curriculum intervention resulted in: ↓ physical activity (*p* = 0.04) Wellness intervention resulted in: ↓ frequency of sweetened beverages (*p* = 0.05) and size (*p* = 0.006) ↓ processed packaged snacks size (*p* = 0.01); candy frequency (*p* = 0.04) ↓ baked good frequency (*p* = 0.05) ↓ fast food frequency (*p* = 0.003), size (*p* = 0.01), and combo meals (*p* = 0.002) Prevalence of overweight and obesity not change Adherence/compliance rates: n.d.	High	***
(44), UK and Canada	Randomized controlled trial	*n* = 54 F overweight/obese adolescents Age, y (mean ± SD): 14.8 ± 2.3 BMI, kg/m^2^ (mean ± SD): 30.2 ± 5.2 (RDa); 29.6 ± 5.0 (LDa); 24.6 ± 2.5 (Control)	12 weeks one-to-one dietary counseling (1 h; 5 sessions): Self-determination theory open questions active listening empathy encourage to “take ownership” of their diet meaningful rationale set specific goals self monitoring specific goals specific informative and non-judgemental feedback support identification of barriers and develop plans, friendly, caring manner, step-count challenges with friends and family 3 groups: – RDa: dairy diet + exercise – LDa: low dairy diet + exercise – Control: no intervention Follow-up: n.d. Professionals: registered dietitian	In RDa group: improvements in body composition RDa and LDa showed significant improvements in: physical self-worth (*p* = 0.001) body satisfaction (*p* = 0.002) perceived physical conditioning (*p* = 0.002) Adherence/compliance rates: RDa 86% and LDa 79%	Low	***
(43), China	Cluster randomized controlled trial	*n* = 814 students *n* = 757 parents Age, y (mean ± SD): 9.3 ± 1.2 (intervention); 9.4 ± 1.2 (control) BMI, kg/m^2^ (mean ± SD): 17.7 ± 3.2 (intervention); 18.4 ± 3.4 (control)	Intervention: Social Cognitive Theory; 4 components targeting children and their parents: – 4 times offline lectures – Nutrition-related manuals and books – 20 health education materials Control: eyes health promotion Follow-up: 12 months Professionals: n.d.	In intervention group: ↑ nutrition knowledge of children and parents no BMI and WHtR reduction Adherence/compliance rates: n.d.	High	***
(45), China	Randomized controlled trial	*n* = 41 students *n* = 26 parents Age, y (mean ± SD): n.d. BMI, kg/m^2^ (mean ± SD): 22.2 ± 2.1 (intervention); 22.1 ± 2.6 BMI z-score (mean ± SD): 1.6 ± 0.3 (intervention); 1.4 ± 0.3 (control)	Intervention ↑ 12 month program: – Nutritional education (60 min monthly) – Exercise intervention (60 min regularly) – Psychological intervention (Social Cognitive Theory) – Fun activity session (during summer 1–2 activities) – Telephone follow-up (every 2 weeks) Control: no intervention Follow-up: n.d. Professionals: n.d.	Intervention resulted: BMI-z score, WHR and WHtR significant improvements NS: ↓ BMI-P, fasting plasma glucose, cholesterol and low-density-lipoprotein cholesterol levels. Adherence/compliance rates: Intervention school: 88.5% Control school: 86.7%	Low	****
(46), Korea	Randomized trial	*n* = 104 children and adolescents with moderate to severe obesity Age, y (mean ± SD): 10.9 ± 2.1 BMI, kg/m^2^ (mean ± SD): n.d. BMI z-score (mean ± SD): 2.3 ± 0.5 (intervention) 2.3 ± 0.5 (control)	24 weeks (6 sessions) Intervention group (NG): nutrition education + one-to-one NC Control group (UG): nutrition education only Follow-up: n.d. Professionals: nutritional expert	In NG: ↓ high-calorie, low-nutrient food consumption ↑ Diet Quality Index-International score ↓ BMI z-score All subjects showed (24 weeks): ↓ energy, carbohydrates, fat, sodium intake no differences between NG and UG Negative association between BMI-z-score and self-efficacy Adherence/compliance rates: n.d.	Low risk	***
**Non-randomized studies**						
(27), USA	Cross-sectional	*n* = 55 children with elevated blood cholesterol Age, y (mean): 11.5 BMI, kg/m^2^ (mean ± SD): n.d.	Social problem-solving skills and activities. Child problem solving ability: “open middle” story completion technique Parent-child interaction: plan a meal separately and after 3 min together to reach an agreement Parenteral facilitation of children problem solving (scale from praised up to punished child) Child’s satisfaction of meal plan (scale 0–100) Child behavior problems (Child behavior checklist) Follow-up: n.d. Professionals: n.d.	Adolescents who were able to generate multiple ways to cope with dietary temptations described in hypothetical vignettes evidenced better dietary adherence than adolescents who could produce fewer coping strategies. Parent-child interaction: child satisfaction with the diet was positively associated with parental attempts to solicit and reinforce the child’s involvement in meal planning. Adherence/compliance rates: dietary adherence *p* < 0.01 (measured as dietary LDLc change)	Some concerns	****
(29), USA	Experimental design consisting of pretest-posttest comparison	*n* = 25 obese adolescents Age, y (mean ± SD): 13.5 ± 0.3 BMI, kg/m^2^ (mean ± SD): 40.1 ± 2.0 BMI z-score (mean ± SD): 2.5 ± 0.1	1-year comprehensive weight-management program: Non-diet, better food choices approach (45 min weekly for 6 of 12-week sessions) Problem solving approach (45 min weekly by a dietitian or social worker for 6 of 12-week sessions) 2 days/week 30-min exercise sessions Diet method subgroups: Dieting group: Structured Meal Plan Non-dieting group: Better Food Choices Follow-up: 2 years Professionals: registered dietitian, social worker	At 1 year: ↓ BMI z score ↓ body fat% ↑ self-concept scores At 2 years: ↓ BMI z score still significant body fat% and self-concept scores remained improved Dietary method: Dieting group tended to show favorable short-term results for BMI z-score at first year (*p* = 0.11); at the second year, the non-dieting group improved BMI z-score (*p* = 0.006), while the dieting group returned toward baseline. Adherence/compliance rates (self-reported, 0 = poor compliance; 2 = high compliance): average > 1	Low risk	****
(28), Switzerland	Experimental design consisting of pretest-posttest comparison groups	*n* = 130 obese children and adolescent Age, y (median; IQR): 13.8; 12.1– 15.0 BMI, kg/m^2^ (median; IQR): 33.4; 30.1–36.6	8-week multidisciplinary inpatient obesity program: nutritional intervention + physical activity program + behavior modification: individual therapy sessions focused on increasing self-esteem, responsibilities, and working on problem-solving strategies Follow-up: n.d. Professionals: nutritionist, exercise therapist	All patients showed: ↓ body weight improvement of all measurements of aerobic fitness ↑ quality of life Adherence/compliance rates: n.d.	Some concerns	***
(30), USA	Experimental design consisting of pretest-posttest comparison	*n* = 23 obese preadolescents with risk for diabetes type 2 Age, y (mean ± SD): 11.7 ± 1.2 BMI, kg/m^2^ (mean ± SD): 33.1 ± 5.9	12-week intervention (social cognitive and self-efficacy theory): 2 weekly physical activity sessions + 4 (45-min) consultations and 2 (60-min) food demonstrations Follow-up: n.d. Professionals: registered dietitian, nurses and physicians	Improvement in physical activity Changes in measures of both task self-efficacy (β = 0.39) and self-regulatory efficacy (β = 0.44) Significant improvement in total cholesterol and BMI Adherence/compliance rates: n.d.	Some concerns	***
(31), USA	Experimental design consisting of pretest-posttest comparison	*n* = 38 African-American students Age, y (mean): 14.9 (Year I); 15.5 (Year II) BMI, kg/m^2^ (mean ± SD): n.d.	6-weeks “Whole School, Whole Community, Whole Child”: collaborative, school-based integrative health promotion. health education positive role models learning healthy substitutes for unhealthy choices self-reevaluation social liberation and empowerment Transtheoretical Model of Health Behavior Change Follow-up: 2 and 8 weeks after the end of the program Professionals: certified personal exercise trainer, yoga and mindfulness instructor + other unspecified figures	Improvements in self-reported physical activity and dietary habits No changes in stressor mindfulness New knowledge and skills ↑ self-efficacy, health behavior change, and program enjoyment Adherence/compliance rates: n.d.	High risk	***
(32), Korea	Quasi-experimental intervention trial	*n* = 103 overweight/obese children Age, y (mean ± SD): 11.7 ± 1.2 (control); 12.9 ± 1.7 (intervention) BMI, kg/m^2^ (mean ± SD): 29.6 ± 4.2 (control); 30.3 ± 4.1 (intervention) BMI z-score (mean ± SD): 2.3 ± 0.5 (control); 2.3 ± 0.5 (intervention)	16-week multidisciplinary lifestyle intervention program (2 groups): Intervention: usual care + exercise Control: usual care only Usual care: one-to-one medical consultation workbook provision for goal setting and behavioral modification exercise counseling physical activity monitoring and feedback one-to-one NC Exercise program (from 5th week): exercise three days/week for 60 min/session (one group exercise session and two home-based exercise sessions) Follow-up: n.d. Professionals: doctors, clinical dietitians, exercise specialists, social workers, nurses	Exercise group showed: ↓ BMI z-score No difference in the BMI z-scores between the usual care and exercise groups after adjustment. Both groups showed: ↑ lean body mass ↓ total energy intake Positive effects on body composition, physical fitness and cardiometabolic risk markers. Adherence/compliance rates: n.d.	Low risk	****
(33), Mexico	Qualitative study	*n* = 564 children teachers, directors, parents and personnel working in the school food store Age, y (mean ± SD): n.d. BMI, kg/m^2^ (mean ± SD): n.d.	Health Communication Process: (1) Theory of cognitive development (2) Social Development Theory (3) Ecological Model Focus groups were conducted as a qualitative technique + Nutritional education Follow-up: n.d. Professionals: psychologists, educators, psychologists, physical educator, nutritionist	The Health Communication Process is an effective tool for program planners to design interventions. Adherence/compliance rates: 100%	Some concerns	***

BMI, body mass index; CHBS, combined health behavior score; DASH, Dietary Approaches to Stop Hypertension; DVD, digital video disc; FV, fruit and vegetable; IQR, interquartile range; NS, non-significative; SD, standard deviation; SSB, sugar sweetened beverages; TV, television; Y, years; WHR, waist-to-hip ratio; WHtR, waist-to-height ratio, LDLc, low-density lipoprotein cholesterol; n.d., no date. ***, ****, *****MMAT quality.

All studies were published between 1990 and 2022 in different countries: United States of America (*n* = 10) (27, 29–31, 34–36, 39, 42, 47), China (*n* = 2) (43, 45), Korea (*n* = 2) (32, 46), Netherlands (*n* = 1) (37), Brazil (*n* = 1) (38), Switzerland (*n* = 1) (28), Mexico (*n* = 1) (33), Italy (*n* = 1) (40). One study was conducted in the United Kingdom and Canada (44) and one study in the USA and Canada (41).

The duration of interventions ranged from 6 weeks up to 1 year. Most of the selected studies (47.6%) focused mainly on the overweight or obese population (28–30, 32, 36, 37, 39, 41, 44, 46). Some of the articles (23.8%) evaluated individuals with chronic diseases [hypertension (35), cystic fibrosis (34, 47), dyslipidemia (27), or risk for diabetes type 2 (30)]. Seven articles (33.3%) were focused on students (31, 33, 38, 40, 42, 43, 45). The interventions were conducted by different figures: multidisciplinary team (*n* = 6) (30, 32–34, 37, 47), nutrition professional (dietitian, nutritionist) (*n* = 5) (35, 38, 41, 44, 46), nutrition professional with another health professional (physician, nurse, exercise therapist or social worker) (*n* = 3) (28, 29, 39), and other figures (*n* = 4) (31, 36, 40, 42). In 3 studies the counselor was not specified (27, 43, 45).

The majority of the studies did not mention any theory to embase the NC (*n* = 10, 47.6%) (28, 29, 32, 34, 35, 38, 40, 41, 46, 47). In the remaining studies, NC interventions were mainly based on: Social Cognitive Theory (*n* = 7, 33.3%) (27, 30, 33, 36, 42, 43, 45), Self Determination Theory (*n* = 3, 14.3%) (30, 42, 44) and Transtheoretical Model of Health Behavior Change (*n* = 2, 9.5%) (31, 39).

The outcomes also varied between studies, some of them reporting several outcomes. Ten studies (47.6%) described anthropometric or body composition changes (28–30, 32, 34, 37, 44–47), six studies (28.6%) investigated dietary intake (31, 35, 38, 40, 41, 46) three studies (14.3%) evaluated nutrition knowledge (36, 39, 43) or physical activity changes, reporting positive (30, 31) or negative results (42). Other outcomes studied were self-efficacy or coping skills (27, 30) and efficacy of a program planners tool to design interventions (33).

Most of the studies (*n* = 12, 57.1%) (28, 30–32, 34–36, 39, 40, 42, 43, 46) did not show adherence or compliance rates for the interventions, while two publications reported lack of adherence (41) or no changes in the adherence or behavioral compliance scale (47). Two articles cited improved compliance as a result of behavioral treatment, but without mentioning the rates (34, 46) mentioned significant results in compliance rates, using low-density lipoprotein cholesterol changes (27) or frequency of food consumption (38). High adherence rates, varying from 59.2 to 100%, were found in 4 studies (33, 37, 44, 45).

**FIGURE 2:**
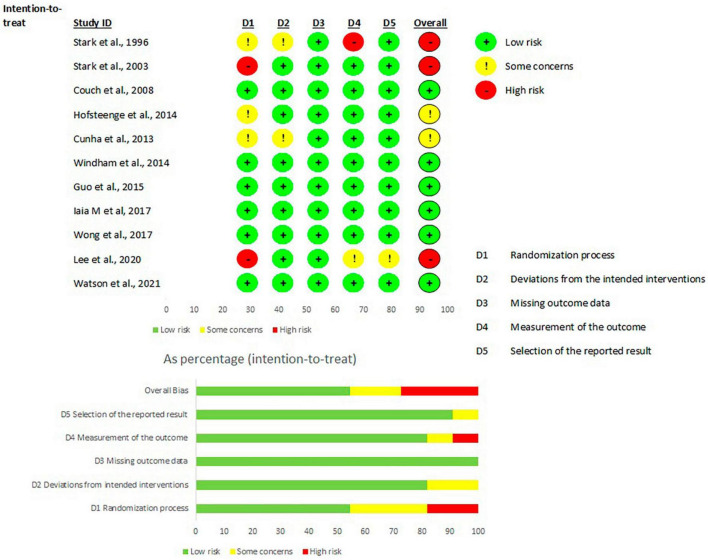
Risk of bias per articles intention-to-treat analysis, according to Rob2 tool. From: Sterne et al. (13).

**FIGURE 3:**
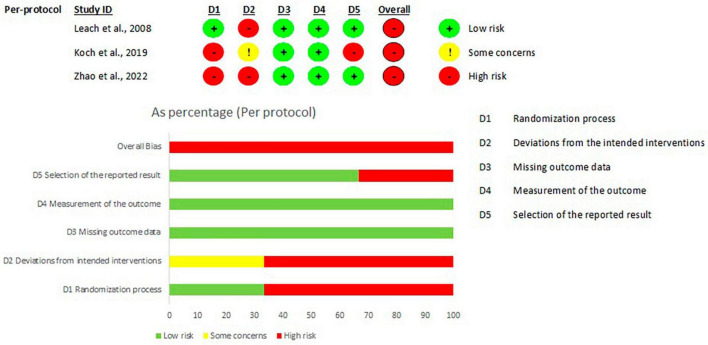
Risk of bias per articles per protocol analysis, according to Rob2 tool. From: Sterne et al. (13).

**FIGURE 4:**
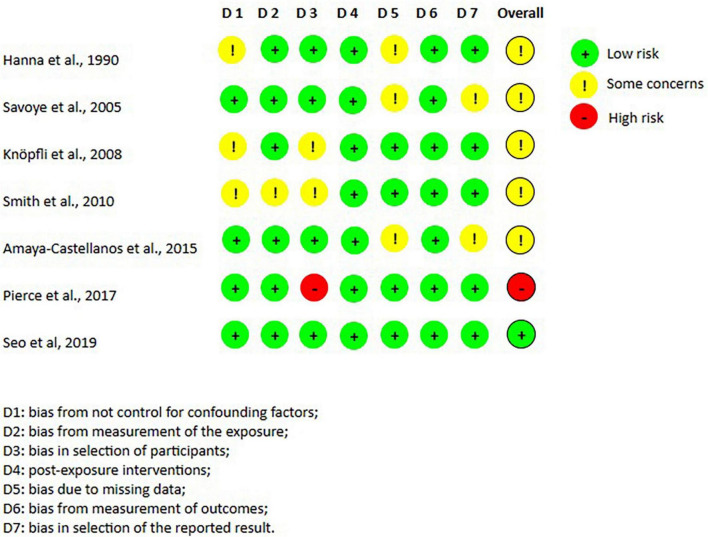
Risk of bias per non-randomized studies, according to Robins tool. From: Created by authors based on Sterne et al. (14).

The randomized trials were divided according to the type of study analysis: 11 intention-to-treat (Figure 2) studies and 3 per-protocol articles (Figure 3). The highest risk of bias for both analyses was in domain 1 (D1), which is related to the randomization process. Conversely, the lowest risk of bias for all studies was in domain 3 (missing outcome data). In domain 4 (i.e., measurement of the outcome), one study presented a high risk of bias (34) and in domain 5 (selection of the reported result) another study (42) presented a high risk of bias. Of the 11 studies included in the intention-to-treat analysis, 6 articles had a low risk of bias, while all the 3 per protocol studies had a high risk of bias. The analysis of the risk of bias for the non-randomized studies (shown in Figure 4) showed that 5 studies had some concerns, mainly because of: (i) a lack of control of all confounding factors (domain 1), (ii) selection of participants (domain 3) and (iii) missing data (domain 5). Only one study presented a low risk of bias (28) and only one (31) was classified with a high risk of bias due to possible bias in the selection of the sample.

The results of the quality of evidence tested by MMAT (15) are reported in Table 3. Ten studies reached three stars (28, 30, 31, 33, 34, 36, 42–44, 46), nine studies (27, 29, 32, 35, 37, 38, 40, 45, 47) were evaluated with four stars, and two studies reached the maximum level of five stars (39, 41).

No bias was detected in analyzing the funding sources from selected articles ((Supplementary Material 1).

### 3.1 Adherence and compliance to NC intervention

Although few articles (*n* = 9) referred to general adherence or compliance rates, some articles reported significant (38), good (29) or great (33) compliance. Adherence and compliance rates ranged from 59% (37) to 100% (33). Otherwise, some studies reported similar compliance rates between intervention and control groups (34, 44, 45) or even lack of adherence (41). Hanna et al. (27) noted that adolescents were able to cope with temptations and better adhere to diet plans.

### 3.2 Nutritional status and body composition

Data regarding nutritional status and body composition were reported in 16 studies (76.2%). Watson et al. (44) described improvements in body composition, while Knöpfli et al. (28) and Stark et al. (34, 47) illustrated a reduction or increase in body weight in patients with cystic fibrosis, respectively. Improvements in BMI were noted in some articles (29, 30, 37, 45, 46), but others did not point out the same benefits (32, 36, 38–40, 42).

### 3.3 Nutritional intake and quality of diet

Energy intake increased in patients with cystic fibrosis (34, 47). Improvements of dietary habits (31) and quality of diet (46) were reported in selected studies. The main improvements were: (i) increased fruit consumption (35, 38, 40), (ii) decreased total fat and/or increase in low fat dairy products consumption (35), (iii) reduced sugared beverages or foods consumption (38, 42) and (iv) water intake increment (41).

### 3.4 Nutrition knowledge

Improvements in nutrition knowledge were reported in some articles (36, 43). Windham et al. (39), described a better comprehension of obesity-related morbidities by parents, while Pierce et al. (31) noted an enhancement in self-efficacy, knowledge and skills in the intervention group.

## 4 Discussion

This systematic review gathered current evidence on the use of NC in childhood and adolescents and emphasized the potential benefits on adherence and compliance, nutrition knowledge, nutritional status and food intake.

Previous reviews about NC in the adult population showed the benefits of this approach. Positive changes in nutrition knowledge and dietary consumption were brought about by NC treatments, which in turn supported individual performance in adult athletes (10). In patients with diseases, such as cancer, NC has been demonstrated to increase protein intake and energy levels (11).

Adolescents are a particular population due to their changing surroundings. A recent scoping review that mapped the heterogeneous literature on NC approach for adolescents found that the effective NC is constructed by multiple NC methods: connecting to the client motivation, providing recurrent feedback, using integrated support tools, showing empathy, including clients preferences, and developing the dietitian’s own professionalism (9).

Our systematic review showed that the overall quality of publications is good, however, improvements still could be made in controlling the confounding factors (non-randomized articles) or during the randomization process (randomized clinical trials), since those are the most common risk of bias found in the selected articles.

The concept of adherence is a topic of continuous discussion in the scientific literature. Terms like compliance and concordance are used interchangeably, despite some evidence supporting their differentiation (48).

The patient-centered techniques associated with NC strategies may theoretically lead to improved adherence; however, this finding was not fully documented in the studies that were included in the analysis. Few studies reported adherence or compliance with heterogeneous methodologies (27, 33, 37, 38, 41, 44, 47). In order to increase the results of an intervention, adherence is a crucial component, and future research should focus more on this area.

In this present review, there are three articles about cystic fibrosis patients. For these patients, some guidelines indicate behavioral intervention strategies associated with nutrition education being more effective when compared to nutritional advice alone. The efficacy of NC was measured by increasing BMI or weight of patients. Currently, NC has been already linked to an improvement in quality of life and wellbeing in pediatric patients with cystic fibrosis (49–52).

Other articles presented the anthropometric parameters improvement in overweight or obese patients by decreasing weight or changing body composition (i.e., reduced body fat% and increased lean body mass). The US Preventive Services Task Force (53) published recommendations for overweight and obese children and adolescents. An extensive and intensive behavioral intervention to promote improvements in weight status was classified as a grade B recommendation. The intensity of the interventions is measured by contact hours. When 26 or more contact hours were used in interventions the results showed an adequate weight status for up to 12 months. The intervention involves multiple components, focused on both the parent and child (separately, together, or both): individual sessions (offered for both family and group); information about healthy eating, safe exercising, and reading food labels. Other strategies like encouraging the use of stimulus control (e.g., limiting access to tempting foods and screen time), goal setting, self-monitoring, contingent rewards, and problem solving; and supervised physical activity sessions were also used (53).

The American Academy of Pediatrics (54) also recently published the clinical practice guidelines for the evaluation and treatment of children and adolescents with obesity. These guidelines support the positive effect of health behavior and lifestyle treatment, based on a child-focused, family-centered approach. The organization recognizes the importance of an intensive intervention focused on health behavior and lifestyle and highlights some of the aspects associated with successful outcomes, some of them also cited as one strategy of NC, such as motivational interviewing (54).

Although one article presented physical activity change in an undesired direction, two studies underlined the importance of NC for developing an active lifestyle. Another systematic review (55), centered on general behavioral strategies, not only necessarily NC, indicated that adding behavioral modifications to the nutritional and physical activity interventions might have an impact on anthropometric outcomes, such as BMI, skinfold thickness, and BMI z scores. This article also stated that family-based therapy, with active parents and children engagement in making healthier choices, is one of the strongest interventions for childhood obesity. If this approach is complemented with Cognitive Behavioral Therapy could be even more beneficial, because the individuals are encouraged to change attitudes and behaviors that support an actual behavior (55).

Nutritional counseling (NC) treatment improved dietary quality in the studied articles, such as increasing fruits and vegetables consumption (35, 38, 40), water intake (41) or reducing total fat (35) or high-calorie, low-nutrient food intake (38, 46).

Nutrition knowledge can directly affect food choices, and consequently health. Childhood is a crucial period to invest in early-behavioral treatments in order to prevent later adult diseases because this first life stage is a particularly susceptible phase for development (56). Even the parent’s nutrition knowledge can influence dietary habits of children (57). In the present review NC has proven to be a good option to supply nutrition knowledge to children, adolescents and their parents (36, 39, 43).

Nutritional counseling (NC) has been used for children and adolescence since 1990 (27), nonetheless it is not yet a standardized terminology. In fact, one limitation of this study is that the search engine did not recognize the term “NC” as a patient-centered behavioral approach, but sometimes the term is misunderstood as simply giving nutritional advice for patients or clients. This limitation can underestimate the number of publications and the results of strings in databases. Another limitation is the heterogeneity of the publications, some interventions for specific diseases, others made in schools, and completely different sample sizes among the included papers. These limitations did not allow to perform a meta-analysis. This paper focuses only on studies with NC direct to the pediatric population. It was not our goal to have NC with parents or schools, nevertheless some studies involved students (31, 33, 38, 42, 43, 45) or done in childcare centers (40), the focus of intervention was the child or adolescent. For that reason, some studies were excluded in the final phase of analysis (16, 17, 20, 58).

There is a burden for pediatric interventions including multi-disciplinary behavioral strategies from NC, since it is important to consider a patient-centered model to change outcomes for the future of generations, independently of the presence of associated diseases. Families, schools, governmental or non-governmental institutions and communities have the duty of providing to all children the access to high-quality, low-cost foods and beverages that are in consonance with life-long healthful eating (8).

In practice, performing NC in pediatrics is challenging. The counseling strategies must focus on adequate content according to the cognitive ability of each age. It is important to emphasize the multidisciplinary of this intervention which must involve pediatricians and also other pediatric healthcare providers [such as registered dietitian nutritionists (RDNs), psychologists, nurses, exercise specialists, and social workers], families, schools, communities, health policy (54). Furthermore, the studies encompassed various diseases, including cystic fibrosis, hypertension, diabetes, and obesity, which necessitate a comprehensive approach to knowledge dissemination and health enhancement. NC is a non-invasive therapeutic approach that ought to be implemented early for nutrition intervention in some disease scenarios (11).

Future studies ought to concentrate on the NC tactics that can be most effective for each age range. In general, effective counseling procedures are described in scientific papers, as opposed to less successful and ineffectual strategies (9). Understanding both successful and unsuccessful tactics may help to enhance interventions and, as a result, produce better health results.

## 5 Conclusion

Nutritional counseling strategies can be effectively used in children and adolescents. Nevertheless, more structured research must be done focused on this population. To invest in good strategies favoring healthy eating behaviors in pediatrics can lead to better health outcomes in the future population with substantial benefits to society.

## Data availability statement

The original contributions presented in this study are included in this article/(Supplementary material, further inquiries can be directed to the corresponding author.

## Author contributions

LN: Conceptualization, Data curation, Investigation, Methodology, Writing – original draft, Writing – review & editing. MG: Investigation, Methodology, Writing – original draft, Writing – review & editing. SF: Conceptualization, Data curation, Investigation, Methodology, Writing – original draft, Writing – review & editing. FQ: Data curation, Investigation, Writing – review & editing. AT: Writing – review & editing. CF: Conceptualization, Investigation, Methodology, Supervision, Writing – original draft, Writing – review & editing.
