# Supplementary material for: Nutritional counseling in childhood and adolescence: a systematic review
Source: Front Nutr. 2024 Feb 1;11:1270048. doi: 10.3389/fnut.2024.1270048 (PMC10867228; doi:10.3389/fnut.2024.1270048)
Supplement: Supplementary file 1 [file Table_1.DOCX]

Supplementary material

S1 - Funding sources for all included articles.

| **Articles** | **Funding/Support** | **Role of the Funder/Sponsor** |
| --- | --- | --- |
| Stark et al., 1996, USA | Supported by a grant from the National Cystic Fibrosis Foundation (no. 2117) to Lori J. Starck |  |
| Stark et al., 2003, USA | / |  |
| Couch et al., 2008, USA | / |  |
| Leach et al., 2008, USA | / |  |
| Hofsteenge et al., 2012, Netherlands | This study is funded by The Netherlands Organisation for Health  Research and Development (ZONMW) (no: 50-50110-98-255). | The funding organization was not concerned with the design and conduct of the study; nor collection, management, analysis, and interpretation of the data; nor preparation, review or approval of the manuscript. |
| Cunha et al., 2013, Brazil | / |  |
| Windham et al., 2014, USA | This project was funded by the Robinson Foundation. |  |
| Iaia et al., 2017, Italy | In September 2012, OROGEL S.p.A., a company based in via  Dismano 2600, I-47522, Cesena, Forlì Cesena, Italy, granted €10 000 in support of our educational intervention. | This sponsor had no role in designing and conducting our study; collecting, managing, analyzing and interpreting its data; and preparing, reviewing and approving our paper. |
| Wong et al., 2017, USA and Canada | This work was conducted with grants from the Airborne Cy Pres Fund and New Balance Foundation and with support from the Harvard Catalyst (Harvard Clinical and Translational Science Center, National Center for Research Resources and the National Center for Advancing Translational Sciences, National Institutes of Health Award UL1 TR001102). DrWong was supported by a Canadian Institutes of Health Research (CIHR) Fellowship Award in the area of Clinical Research and a CIHR Randomised Controlled Trials—Mentoring Program Training Grant. Dr Ludwig was supported by a mid-career mentoring award from the National Institute of Diabetes and Digestive and Kidney Diseases (K24 DK082730). | The funding sources had no role in the design and conduct of the study; collection, management, analysis, and interpretation of the data; preparation, review, or approval of the manuscript; and decision to submit the manuscript for publication. |
| Koch et al., 2019, USA | This study was funded by US Department  of Agriculture, Agriculture and Food Research Initiative, Human Nutrition and Obesity, Grant/Award No. 2010-85215-20661. |  |
| Watson et al., 2021, UK and Canada | This work was supported by funding from Dairy Farmers of Canada. Dairy products were kindly provided by Parmalat (milk and cheese) and Danone (Greek Yoghurt). | Disclosure of potential conflicts of interest: Dr Josse reports grants from Dairy Farmers of Canada, non-financial support from Danone, non-financial support from Parmalat, during the conduct of the study; personal fees from Dairy Farmers of Canada Grant Review Board, outside the submitted work. |
| Zhao et al., 2022, China | This work was supported in part by the Chinese Nutrition Society (grant number CNS-NNSRG2019–97) |  |
| Guo et al., 2015, China | / |  |
| Lee et al., 2020, Korea | This study was funded by the Korea Centers for Disease Control  and Prevention (grant number: 2016-ER6405-00). | The sponsors had no role in the design of the study; in the writing of the manuscript, or in the decision to publish the results. |
| Hanna et al., 1990, USA | The research was supported in part by grant ROl-HL36298 from the National Heart, Lung and Blood Institute to Craig K. Ewart and by the Lipid Clinic of the Division of Pediatric Medicine, Department of Medicine, Johns Hopkins Hospital. |  |
| Savoye et al., 2005, USA | This work was supported by grants from National Institutes of Health (NIH) grants RO1-HD28016, NIH M01-RR06022, NIH M01-RR00125, and T32 NR008346. |  |
| Knöpfli et al., 2008, Switzerland | / |  |
| Smith et al., 2010, USA | This research was supported by the Friends Research Fund of Children's Healthcare of Atlanta, Atlanta, GA. |  |
| Pierce et al., 2017, USA | This intervention and outcomes evaluation was funded by the Institute for Integrative Health, the Zanvyl and Isabelle Krieger fund, and the National Institutes of Health, USA (T35 DK095737) |  |
| Seo et al., 2019, Korea | This research was funded by the Korea Centers for Disease Control and Prevention, grant number 2015-ER6401-00. |  |
| Amaya-Castellanos et al., 2015, Mexico | This study was supported by the State System for the Comprehensive Development of the Family, State of Mexico (DIFEM). |  |
